# Influence of time to diagnosis of severe influenza on antibiotic use, length of stay, isolation precautions, and mortality: a retrospective study

**DOI:** 10.1111/irv.12454

**Published:** 2017-05-12

**Authors:** Isabel E. Akers, Rainer Weber, Hugo Sax, Jürg Böni, Alexandra Trkola, Stefan P. Kuster

**Affiliations:** ^1^ Division of Infectious Diseases and Hospital EpidemiologyUniversity Hospital and University of ZurichZurichSwitzerland; ^2^ Institute of Medical VirologyUniversity of ZurichZurichSwitzerland

**Keywords:** antibiotic use, influence of time to diagnosis, influenza

## Abstract

**Background:**

Timely diagnosis of influenza infection in patients might help reduce antibiotic use during influenza seasons and, consequently, antibiotic selection pressure. In this retrospective cohort study, we aimed to evaluate whether time to influenza diagnosis in patients with severe influenza is associated with the duration of antibiotic therapy.

**Methods:**

We retrospectively included all hospitalized patients >16 years who tested positive for influenza A or B by polymerase chain reaction during influenza seasons 2013/2014 or 2014/2015 at the University Hospital Zurich. The primary aim was to assess the association between timing of laboratory‐confirmed influenza diagnosis and duration of antibiotic therapy. Secondary outcomes were length of hospital stay, duration of isolation precautions, and mortality. Early diagnosis was defined as laboratory confirmation on the day of or the day after hospital admission or symptom onset.

**Results:**

A total of 126 patients were included (median age 57 years). Timing of influenza diagnosis was not associated with the duration of antibiotic treatment, the duration of isolation precautions, or mortality. Early influenza was associated with reduced length of hospital stay (median 7 vs 9 days [*P*=.014]) in patients with community‐acquired influenza.

**Conclusions:**

Although the duration of antibiotic therapy and mortality were found unaffected by early influenza diagnosis, our data indicate that it is linked with a reduction in the length of hospitalization in patients with community‐acquired influenza. This highlights a need to also fully understand the effect of time to diagnosis of bacterial pathogens on antibiotic prescribing patterns in order to exploit the potential of early influenza diagnosis in patient care.

## 1 Introduction

Unspecific symptoms hamper influenza diagnosis based on clinical case presentation alone and differential diagnoses can be quite diverse, misinterpreting influenza infections as common cold, bacterial pneumonia or even non‐infectious causes of fever.^1^, ^2^ Although the likelihood of influenza is increased during winter season in patients with cough and fever,^1^, ^3^, ^4^ these predictors do not enable physicians to differentiate adequately between the various differential diagnoses.

Due to these unspecific and often misleading symptoms and potentially severe complications, hospitalized patients with suspected or confirmed influenza diagnosis often receive antibiotic treatment.^5^, ^6^ Recommendations are to base influenza treatment on neuraminidase inhibitors together with symptomatic therapy and to use antibiotics only in severe cases or patients with high risks.^7^, ^8^, ^9^ In severe cases, an antibiotic agent active against *Staphylococcus aureus* is recommended, as staphylococcal pneumonia is known to be frequently associated with influenza.^10^ Interestingly, even though early influenza treatment with neuraminidase inhibitors was previously shown to be associated with shorter duration and reduced severity of illness, a faster resolution of fever, and a faster return to normal health and activity,^11^, ^12^ only 1.6% of patients with influenza‐like symptoms in season 2014/2015 were treated with neuraminidase inhibitors in Switzerland, and even less (0.4%) in 2013/2014.^13^ This management strategy, however, might also be influenced by study results that show only moderate benefit of neuraminidase inhibitors in reducing influenza symptoms in healthy outpatients and the lack of controlled clinical trials supporting a beneficial effect on severe outcomes in hospitalized patients despite a growing body of evidence from observational studies.^14^, ^15^, ^16^, ^17^

A recent survey highlights that up to date, antiviral substances are underutilized and antibiotics overused in influenza patients.^5^ Despite the fact that in hospitalized adults with acute respiratory illness during winter season, viral infection seems to be more likely than bacterial,^6^ patients often receive antibiotic treatment despite a diagnosis of respiratory virus infection.^18^

More rapid influenza diagnosis may thus help streamline anti‐infective treatment and reduce antibiotic consumption. As antibiotic overuse is associated with emergence of antibiotic resistance,^19^ rapid influenza diagnosis may therefore reduce antibiotic selection pressure, resistance development, and support appropriate treatment. Consequently, rapid diagnosis of influenza has previously been shown to help reduce the use of antibiotics in adult outpatients^20^ and was shown to reduce the duration of antibiotic therapy, the amount of prescription, and the length of hospital stay in children.^21^, ^22^ Recent studies suggested a general benefit of hospitalized patients from early influenza diagnosis by reducing the need for extended care after hospital discharge in older adults,^23^ mortality,^24^ and the risk of lower respiratory tract complications.^25^ Oosterheert et al. and Shiley et al. reported that viral respiratory tract diagnosis *per se* does not impact the use and duration of antibiotic treatment in hospitalized adults.^18^, ^26^ In contrast, Rogers et al. showed that rapid influenza diagnosis did decrease duration of antibiotic use, length of hospitalization, and duration of isolation in children admitted with respiratory symptoms.^21^

To clarify and validate the benefits of early diagnosis, we aimed to determine whether rapid laboratory confirmation of influenza infection in hospitalized adults reduced the duration of antibiotic therapy in a retrospective cohort study of adults with influenza diagnosis hospitalized at the University Hospital Zurich, Switzerland, in seasons 2013/2014 and 2014/2015. Secondary aims of our study were the analysis of the association between timeliness of influenza diagnosis, length of hospital stay, duration of isolation precautions, and in‐hospital mortality.

## 2 Materials and Methods

### 2.1 Study setting, design, and procedures

We performed a single‐center retrospective cohort study of hospitalized patients with laboratory‐confirmed influenza diagnosis at the University Hospital Zurich over the 2013/2014 and 2014/2015 influenza seasons, defined as the time between the first and the last positive influenza test result in our institution in the time period between October 1 and April 30. The University Hospital Zurich is a 900‐bed university‐affiliated tertiary care center that covers all specialties except orthopedic surgery and pediatrics. It serves a population of 400 000 inhabitants for primary and 1 443 000 for tertiary care. Approximately 540 000 outpatients and 38 000 inpatients receive medical treatment each year.^27^, ^28^, ^29^

Our study retrospectively included all hospitalized and consenting patients greater than 16 years of age with influenza A or B infection confirmed by polymerase chain reaction (PCR)^30^ from respiratory specimens (nasopharyngeal swab or broncho‐alveolar lavage) in influenza seasons 2013/2014 and 2014/2015. Patients were identified from the infection control database, where all patients with suspected and confirmed influenza diagnosis are recorded. Clinical data (including baseline patient characteristics, living situation, influenza‐like symptoms, vital signs and clinical findings upon admission or symptom onset, admission diagnoses, laboratory sampling, and disease treatment details) were then extracted from electronic medical records with the use of a standardized questionnaire and collected in an electronic database (Microsoft Access^®^, Version 14.0.7151.5001, 2010, Microsoft Corporation, Redmond, WA, USA). The pneumonia severity index (PSI),^31^ the Charlson comorbidity index,^32^ and the CURB‐65 Score^31^ were used to classify the severity of respiratory disease. “Generalized weakness” was defined as feeling weaker than before onset of symptoms.

### 2.2 Definitions

#### 2.2.1 Predictor variable

The main predictor variable was the duration between symptom onset or hospital admission (whichever occurred first) and laboratory confirmation of influenza. Early influenza diagnosis was defined as laboratory confirmation on the day of or the day after hospital admission in patients with community‐acquired influenza infection and the day of or the day after symptom onset in patients with nosocomial influenza infection, respectively. Nosocomial influenza was defined as occurrence of the first symptoms after admission to the hospital.

#### 2.2.2 Outcome variables

Duration of antibiotic therapy was the primary outcome variable of interest. To estimate the duration of antibiotic therapy, all antimicrobials with antibacterial activity were included if treatment was initiated after the first symptom and stopped after laboratory confirmation of influenza infection. The variable beta‐lactam antibiotics was divided into a broad‐spectrum group with (including piperacillin, cefepime, ceftazidime, meropenem, and imipenem) and a narrow‐spectrum group without (including penicillin, amoxicillin, amoxicillin/clavulanic acid, cefuroxime, cefotaxime, ceftriaxone, ertapenem) antipseudomonal activity.

Secondary outcomes were length of hospital stay, duration of isolation precautions and in‐hospital mortality. Length of stay was defined as time between admission and discharge. Associations with length of hospital stay were analyzed for patients with community‐acquired influenza infection only.

### 2.3 Statistical analysis

Categorical data were tested for differences using Fisher exact tests, whereas continuous variables were tested using Wilcoxon rank sum tests or the Student's *t* test, as appropriate. Cox regression analysis was used to detect differences in length of antibiotic therapy and length of hospital stay in patients with early as compared to late influenza diagnosis. Multivariable logistic regression analysis was used to determine differences in mortality. Potential confounders among patient characteristics with *P‐*values <.05 in univariable analyses were considered for inclusion in multivariable models based on clinical judgment, with final models representing those that best balanced parsimony and fit. The limited number of outcomes was factored in when building the models to prevent overfitting.^33^ Data were analyzed using Stata ^®^ version 13.1 (Stata Corporation, College Station, TX, USA). Two‐tailed *P*‐values <.05 were considered statistically significant.

### 2.4 Ethics

The research was conducted in accordance with the Declaration of Helsinki and national and institutional standards. The study was reviewed and approved by the ethics committee of the Canton of Zurich (BASEC‐Nr.: PB_2016‐00182). A general consent was given by the ethics committee for all patients hospitalized before January 1, 2014, and for deceased patients, tourists, or patients who could not be contacted by mail or phone. Informed consent was obtained from all patients that were hospitalized with influenza at or after January 1, 2014.

## 3 Results

In total, 126 consenting patients with positive influenza PCR could be included in the study cohort. Fifty‐three (42%) were diagnosed with influenza on the day of or the day after admission or symptom onset (early diagnosis group), respectively. Patient characteristics and outcomes are shown in Tables 1 and 2 and S1.

**Table 1:** Patient characteristics in a retrospective cohort of 126 hospitalized patients with laboratory‐confirmed influenza diagnosis during influenza seasons 2013/2014 and 2014/2015

Characteristic	Early influenza diagnosis (n=53)	Late influenza diagnosis (n=73)	*P*‐value
Age, years, median (range)	58 (21‐ 93)	61 (16‐92)	.33
Male sex, n (%)	28 (52.8)	38 (52.1)	1.00
Symptoms upon influenza diagnosis			
Fever, n (%)	37 (69.8)	45 (61.6)	.45
Cough, n (%)	37 (69.8)	57 (78.1)	.31
Dyspnea, n (%)	20 (37.7)	24 (32.9)	.58
Rhinitis, n (%)	12 (22.6)	10 (13.7)	.24
Laryngitis, n (%)	9 (17.0)	19 (26.0)	.28
Headache, n (%)	9 (17.0)	17 (23.3)	.51
Generalized weakness, n (%)	8 (15.1)	3 (4.1)	.05
Shivering, n (%)	7 (13.2)	13 (17.8)	.62
Myalgia or arthralgia, n (%)	7 (13.2)	8 (11.0)	.78
Vomiting or nausea, n (%)	5 (9.4)	7 (9.6)	1.00
Fatigue, n (%)	4 (7.6)	5 (6.8)	1.00
Chest pain, n (%)	3 (5.7)	2 (2.7)	.65
Flank pain, n (%)	1 (1.9)	2 (2.7)	1.00
Back pain, n (%)	1 (1.9)	2 (2.7)	1.00
Diarrhea, n (%)	1 (1.9)	8 (11.0)	.08
Temperature			
Admission or first symptom, °C, median (range)	38.6 (38‐40)	38.6 (38‐40.4)	.62
Maximum, °C, median (range)	39.1 (38‐40.1)	38.8 (38‐40.9)	.82
Blood pressure on admission or first symptom			
Systolic, mm Hg, median (range)	131 (53‐220)	120 (68‐168)	.17
Diastolic, mm Hg, median (range)	77 (40‐103)	67 (35‐120)	.045
Total leukocyte count			
Admission or first symptom, G/L, median (range)	7.52 (0.6‐ 24.69)	5.92 (0.3‐32.54)	.008
Maximum, G/L, median (range)	9.85 (0.98‐28.18)	8.92 (1.13‐68.44)	.36
Total lymphocyte count			
Admission or first symptom, G/L, median (range)	0.62 (0.03‐ 4.83)	0.62 (0.02‐2.65)	.78
Maximum, median G/L (range)	1.65 (0.27‐4.96)	1.64 (0.25‐ 11.84)	.71
Neutrophil count			
Admission or first symptom, G/L, median (range)	5.67 (0.01‐18.83)	4.25 (0.01‐30.22)	.006
Maximum, G/L, median (range)	7.39 (0.33‐24.03)	6.94 (0.18‐62.11)	.42
C‐reactive protein (mg/L)			
Admission or first symptom, mg/L, median (range)	55 (3.8‐440)	59.5 (0.3‐536)	.98
Maximum, mg/L, median (range)	98 (12‐ 440)	138 (4.1‐536)	.42
Charlson comorbidity index on admission or first symptom, median (range)	4 (0‐12)	5 (0‐12)	.08
Pneumonia severity index on admission or first symptom, median (range)	89 (24‐222)	88 (9‐197)	.43
CURB‐65 Score on admission or first symptom, median (range)	1 (0‐5)	1 (0‐ 4)	.38
Admission diagnosis			.00
Pneumonia, n (%)	12 (22.6)	7 (9.5)	<.001
Influenza, n (%)	4 (7.6)	1 (1.4)	
Flu‐like illness, n (%)	10 (18.9)	2 (2.7)	
Asthma exacerbation, n (%)	0 (0)	1 (1.4)	
COPD exacerbation, n (%)	4 (7.6)	3 (4.1)	
Exacerbation of cystic fibrosis, n (%)	2 (3.8)	0 (0)	
Sepsis/SIRS, n (%)	6 (11.3)	18 (24.7)	
Bronchitis, n (%)	1 (1.9)	0 (0)	
Other, n (%)	14 (26.4)	41 (56.2)	
Community‐acquired infection, n (%)	46 (86.8)	51 (69.9)	.032

COPD, chronic obstructive pulmonary disease.

**Table 2:** Outcomes of hospitalized patients with laboratory‐confirmed influenza diagnosis during influenza seasons 2013/2014 and 2014/2015

Outcome	Early influenza diagnosis (n=53)	Late influenza diagnosis (n=73)	*P*‐value
Duration of antibiotic therapy, days, median (range)	5 (0‐45)	7 (0‐57)	.27
Any antibiotic therapy, n (%)	38 (71.7)	57 (78.1)	.53
Length of hospital stay, days, median (range)	7 (2‐58)	9 (3‐95)	.058
In‐hospital death, n (%)	3 (5.7)	6 (8.2)	.73
Isolation precautions			
Droplet precautions, n (%)	51 (96.2)	68 (93.2)	.7
Preemptive isolation after sampling, n (%)	8 (15.1)	4 (5.5)	.17
Isolation after influenza diagnosis, n (%)	43 (81.1)	64 (87.7)	
No isolation, n (%)	2 (3.8)	5 (6.8)	
Duration of isolation precautions, days, median (range)	6 (1‐23)	8 (2‐39)	.29
Antibiotic therapy			
Narrow‐spectrum beta‐lactam, n (%)	21 (39.6)	14 (19.2)	.015
Antipseudomonal beta‐lactam, n (%)	17 (32.1)	31 (42.5)	.27
Macrolide	19 (35.9)	7 (9.6)	.001
Fluoroquinolone	2 (3.8)	12 (16.4)	.041
Antiviral therapy			
Any antiviral therapy	36 (67.9)	39 (53.4)	.14
Preemptive treatment, n (%)	9 (17.0)	14 (19.2)	.82
Initiation after influenza diagnosis, n (%)	27 (50.9)	25 (34.3)	.07
Treatment duration, days, median (range)	7 (1‐54)	7 (2‐24)	.43

ICU, intensive care unit.

Twenty‐nine (23%) patients acquired influenza through nosocomial infection. Nosocomial influenza occurred after a median (range) of 8 (1‐45) days of hospitalization. Primary admission diagnoses of these 29 patients were cardiological (11 [37.9%] patients), hematological (6 [20.7%]), gastrointestinal (5 [17.2%]) and other (7 [24.1%]) disorders. Patients with nosocomial influenza were older (median [range] age: 65 [36‐93] years vs 58 [16‐86] years, *P*=.016) and had a higher Charlson comorbidity index (median [range] 5 [1‐9] vs 4 [0‐12], *P*=.019) and a higher PSI (median [range] 97 [47‐197] vs 87 [9‐222], *P*=.024) than patients with community‐acquired influenza. In addition, they were more likely to exhibit fatigue (6 [20.7%] patients vs 3 [3.1%] patients, *P*=.005), had lower leukocyte counts (median [range] 5.47 [0.06‐24.69] G/L vs 7.01 [0.03‐32.54] G/L, *P*=.042), and lower neutrophil counts upon first symptom onset (median [range] 3.71 [0.01‐14.01] G/L vs 5.18 [0.01‐30.22] G/L, *P*=.045) than patients with community‐acquired influenza on admission, but had higher maximum C‐reactive protein values (median [range] 210 [27‐484] mg/L vs 103 [4.1‐536] mg/L, *P*=.018).

According to univariable analysis, patients with delayed influenza diagnosis had lower total neutrophil counts (*P*=.006), lower total leukocyte counts (*P*=.008), lower diastolic blood pressure on admission/symptom onset (*P*=.045), and were more likely to have acquired influenza infection nosocomially (*P*=.032) than patients with early influenza diagnosis.

### 3.1 Duration of antibiotic therapy

**Figure 1:**
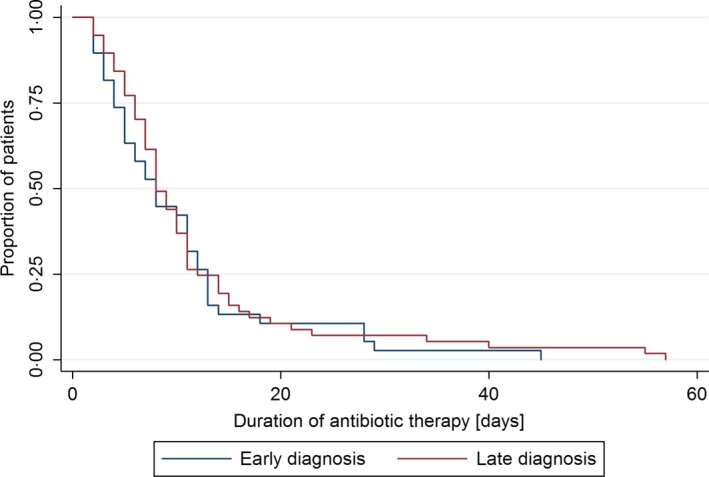
Kaplan‐Meier curve for duration of antibiotic therapy in hospitalized patients with laboratory‐confirmed influenza diagnosis during influenza seasons 2013/2014 and 2014/2015

Ninety‐five of 126 patients (75.4%) received antibiotic therapy, 38 of 53 (71.7%) of the early influenza diagnosis group, and 57 of 73 (78.1%) of the late influenza diagnosis group (*P*=.53). The median (range) duration of antibiotic therapy was 5 (0‐45) days in the early and 7 (0‐57) days in the late diagnosis group. Multivariable Cox regression analysis did not show a difference in the duration of antibiotic therapy (adjusted hazard ratio (HR): 2.4, 95% confidence interval (CI): 0.86‐6.70, *P*=.09; unadjusted HR: 0.89, 95% CI: 0.59‐1.34) between the early and late influenza diagnosis group after adjustment for generalized weakness, bilateral vs unilateral chest X‐ray findings, and treatment with beta‐lactam antibiotics with antipseudomonal activity (Figure 1). Generalized weakness on admission/symptom onset (*P*=.023) and unilateral vs bilateral chest X‐ray findings (*P*=.023) were independently associated with a shorter duration of antibiotic therapy.

### 3.2 In‐hospital mortality

Nine of 126 patients (7.1%) died during hospitalization. Late influenza diagnosis was not associated with in‐hospital death in univariable logistic regression analysis (odds ratio [OR]: 1.50, 95% CI: 0.36‐6.26, *P*=.58) (Table 3).

**Table 3:** Univariable analysis of risk factors for in‐hospital death

Risk factor	Survivors (n=117)	Deaths (n=9)	*P*‐value^a^	Odds ratio (95% confidence interval)^a^
Early influenza diagnosis, n (%)	50 (42.7)	3 (33.3)	.73	1.50 (0.36‐6.26)
Community‐acquired influenza infection, n (%)	91 (77.8)	6 (66.7)	.43	1.75 (0.41‐7.48)
ICU admission, n (%)	34 (29.1)	7 (77.8)	.005	8.54 (1.69‐43.23)
Mechanical ventilation, n (%)	16 (13.7)	6 (66.7)	.001	12.63 (2.87‐55.62)
Charlson comorbidity index, median (range)	4 (0‐12)	6 (3‐12)	.020	1.32 (1.05‐1.66)
CURB‐65 score, median (range)	1 (0‐4)	1 (0‐5)	.047	1.94 (1.16‐3.23)
Pneumonia severity index, median (range)	88 (9‐175)	123 (71‐222)	.017	1.93 (1.01‐1.05)
C‐reactive protein				
Admission, mg/L, median (range)	52 (2.6‐440)	113.5 (0.3‐536)	.019	1.01 (1.00‐1.01)
Maximum, mg/L, median (range)	109 (4.1‐484)	250 (27‐536)	.015	1.01 (1.00‐1.01)
Maximum total neutrophil count, G/L, median (range)	6.9 (0.18‐62.11)	19 (2.78‐37.14)	.036	1.07 (1.02‐1.13)
Diastolic blood pressure on admission or first symptom, mm Hg, median (range)	73 (35‐120)	54 (39‐67)	<.001	0.91 (0.86‐0.96)

According to Fisher exact tests or Wilcoxon rank sum tests, as appropriate.

a Per unit increase (for continuous variables in univariable logistic regression analysis).

ICU, intensive care unit.

### 3.3 Length of hospital stay

**Figure 2:**
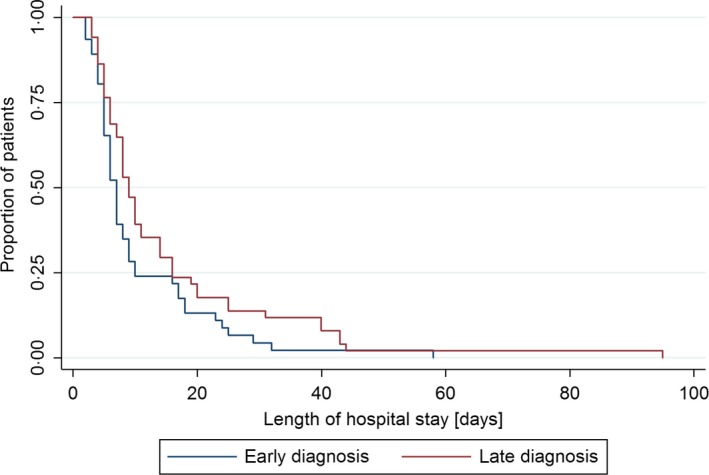
Kaplan‐Meier curve for length of hospital stay in hospitalized patients with laboratory‐confirmed influenza diagnosis during influenza seasons 2013/2014 and 2014/2015

Ninety‐seven of 126 (77.0%) influenza infections were community‐acquired, 46 of 53 (86.8%) in the early diagnosis group and 51 of 73 (69.9%) in the late diagnosis group (*P*=.032). In multivariable Cox regression analysis of the subgroup with community‐acquired infection, the duration of hospitalization was shorter in patients with early influenza diagnosis (adjusted HR: 0.51, 95% CI: 0.30‐0.88, *P=*.014; unadjusted HR: 0.70, 95% CI: 0.47‐1.06; Figure 2) after adjustment for C‐reactive protein level at admission, isolation precautions, and treatment with beta‐lactam antibiotics with antipseudomonal activity.

### 3.4 Isolation precautions

Droplet precautions were initiated in 119 (94.4%) patients, 51 of 53 (96.2%) in the early influenza diagnosis group, and 68 of 73 (93.2%) in the late diagnosis group (P=.70). Most (84.9%) patients were only isolated after influenza diagnosis. The timing of influenza diagnosis was not associated with the duration of isolation precautions (*P*=.17).

## 4 Discussion

Influenza‐like symptoms are common reasons for outpatient visits during influenza seasons. Records refer to approximately 2500 visits per 100 000 inhabitants per season in Switzerland over the last 10 years.^13^ The 2014/2015 influenza season was comparatively severe, with around 3400 consultations per 100 000 inhabitants, and four times more patients with an influenza diagnosis were hospitalized in the 2014/2015 season (4709 [0.8%] as compared to an average of 1173 of hospitalizations per season in the previous 4 years).^13^ Overall, influenza accounts for 1.5% of all hospitalizations due to respiratory diseases in Switzerland per year.^34^ In comparison, around one million patients are hospitalized with primary pneumonia or influenza in the United States annually, representing 12.4% of the primary respiratory and circulatory hospitalizations.^35^

In a retrospective cohort study of hospitalized patients with influenza infection during the 2013/2014 and 2014/2015 influenza seasons in a tertiary care university‐affiliated hospital in Zurich, Switzerland, we found that rapid influenza diagnosis was not associated with a reduction in the duration of antibiotic use, nor with a difference in mortality. However, length of hospital stay was shown to be shorter in patients with community‐acquired influenza infection and early influenza diagnosis. The proportion of isolation precautions and the duration of isolation were not different between patients with early and late influenza diagnosis, and only a small proportion of patients was preemptively put under isolation precautions.

We hypothesized that early diagnosis would reduce antibiotic use and antibiotic treatment duration in hospitalized patients with influenza diagnosis, as viral respiratory infections normally do not require antibiotic therapy. This hypothesis was supported by Jeong et al., who showed a reduction of antibiotic prescriptions in outpatients with flu‐like symptoms after introduction of an influenza virus rapid antigen test in the emergency department.^20^ Furthermore, Rogers et al. demonstrated that rapid influenza detection reduces the use of antibiotics in hospitalized children.^21^ Our study, however, could not confirm such an effect on the duration of antibiotic therapy. Presumably, this was due to the severity of illness in our inpatient population. In Jeong et al.'s study, the patients were not hospitalized, and the study of Rogers et al. was limited to children, which normally do not have many comorbidities. Our study, on the other hand, included hospitalized and mostly comorbid patients, and antibiotic therapy in these patients is in line with current recommendations to empirically treat for presumed superinfection by bacterial pathogens.^36^, ^37^

Despite the fact that there is growing evidence that early antiviral treatment may lower influenza‐associated mortality in hospitalized patients,^24^ only a small proportion of patients of our cohort were empirically treated with neuraminidase inhibitors, and we could not detect a difference in antiviral treatment patterns between patients with early and late influenza diagnosis. Less than 60% of all patients with severe influenza diagnosis received appropriate treatment with neuraminidase inhibitors, indicating a need for improvement of physicians’ prescribing behaviors. Recent findings suggest that the initiation of antiviral treatment after even more than 48 hours of symptom onset in patients with severe influenza is associated with improved survival.^17^

Low mortality rates and thus lack of statistical power may have contributed to the failure of our study to detect any association between time of diagnosis and mortality. Nevertheless, statistical power was sufficient to confirm known predictors of in‐hospital death, such as Charlson comorbidity index, ICU admission, the use of broad‐spectrum antibiotics, and the need of mechanical ventilation.

The association between early influenza diagnosis and a reduced length of hospital stay is in line with the findings by Rogers et al.^21^ and Chaves et al.^23^, ^38^ The reasons for this finding remain unclear, although explanations could be a doctor's delay in unclear or more severe cases without typical signs and symptoms of influenza infection or late initiation of antiretroviral treatment resulting in increased duration of hospitalization. Other studies demonstrated that early treatment is associated with shorter duration and alleviation of symptoms,^11^, ^39^ and a reduction of extended care after hospitalization.^23^, ^38^ Our study, however, in which antiviral use was low, is not able to confirm or rebut any of these findings.

Our study failed to demonstrate an effect of early diagnosis on the duration of isolation precautions. Even though our study population is not suitable for determining the effect of early diagnosis on days of isolation in patients with suspected—rather than confirmed—influenza diagnosis, such an effect may be small in our setting as preemptive isolation precautions were only taken in a small fraction of patients with influenza diagnosis.

Our study has several limitations. First, the findings of our study may not be generalizable to other settings. The University Hospital Zurich is a tertiary care referral center where, because of its many highly specialized departments, many patients with severe comorbidities, such as lung and heart transplant recipients, or oncological patients receive treatment. The patients included in this study may thus not represent a cohort of hospitalized influenza patients as seen in other hospitals. Nevertheless, our patient population represents those at highest risk for severe influenza complications and therefore is of particular interest. Our findings may also not be translated to other influenza seasons or other regions in the same influenza seasons due to antigenic drift and changes in predominant influenza strains over time and even between geographically distinct settings. Second, our observations are only applicable to hospitalized adults with severe influenza; they cannot be extrapolated to adult outpatients with mild influenza or children. Early diagnosis might help optimize treatment and influence antibiotic selection pressure in these populations, as shown by others.^20^, ^21^, ^22^, ^23^, ^24^, ^25^, ^38^ Last, the interpretation of our findings is limited by the retrospective design of the study. Our results should be confirmed in the setting of a prospective study to eliminate bias.

In conclusion, we were able to demonstrate that diagnosis on the day of or the day after admission in hospitalized patients with community‐acquired influenza infection is associated with shorter hospitalization. In contrast, timely diagnosis did not influence the duration of antibiotic therapy, the duration of isolation precautions, or mortality in hospitalized patients with influenza. Physicians should consider early testing for influenza in patients with respiratory symptoms as this may facilitate earlier discharge and to re‐assess the need for antibiotic treatment. Further research should focus on the timeliness of diagnosis of not only influenza but also other respiratory pathogens and its influence on antibiotic use for situations where viral respiratory pathogens in the absence of bacterial superinfections are detected.^33^

## Conflict of Interest

None.
