# Supplementary material for: Influence of time to diagnosis of severe influenza on antibiotic use, length of stay, isolation precautions, and mortality: a retrospective study
Source: Influenza Other Respir Viruses. 2017 May 12;11(4):337–44. doi: 10.1111/irv.12454 (PMC5485868; doi:10.1111/irv.12454)
Supplement: Supplementary file 1 [file IRV-11-337-s001.docx]

**Supplementary table 1:**

Additional patient characteristics in a retrospective cohort of 126 hospitalized patients with laboratory-confirmed influenza diagnosis during influenza seasons 2013/2014 and 2014/2015

| **Characteristic** | **Early influenza diagnosis**  (n=53) | **Late influenza diagnosis**  (n=73) | ***P*-value** |
| --- | --- | --- | --- |
| Family members with respiratory symptoms, n (%) | 5 (9.4) | 4 (5.5) | 0.49 |
| Children in the same household, n (%) | 3 (5.7) | 3 (4.1) | 0.70 |
| Travelling in two weeks prior to diagnosis, n (%) | 3 (5.7) | 2 (2.7) | 0.65 |
| Auscultation findings on admission or first symptom |  |  |  |
| Vesicular, n (%) | 19 (35.8) | 29 (39.7) | 0.55 |
| Crackles, n (%) | 20 (37.7) | 17 (23.3) | 0.15 |
| Wheeze, n (%) | 9 (17.0) | 13 (17.8) | 1.00 |
| Rhonchi, n (%) | 8 (15.1) | 3 (4.1) | 0.05 |
| Pathological findings on auscultation on admission or first symptom |  |  |  |
| Unilateral, n (%) | 6 (11.3) | 7 (9.5) | 0.56 |
| Bilateral, n (%) | 20 (37.7) | 22 (30.1) |  |
| No pathological auscultation | 27 (50.9) | 44 (60.3) |  |
| Chest X-ray results on admission or first symptom |  |  |  |
| Lobar pneumonia, n (%) | 2 (3.8) | 6 (8.2) | 0.47 |
| Interstitial pneumonia, n (%) | 0 (0) | 1 (1.4) | 1.00 |
| Bronchopneumonia, n (%) | 2 (3.8) | 3 (4.1) | 1.00 |
| Pleural effusion, n (%) | 8 (15.1) | 14 (19.2) | 0.81 |
| Pathological findings on chest X-ray on admission or first symptom |  |  |  |
| Unilateral, n(%) | 6 (11.3) | 5 (6.8) | 0.71 |
| Bilateral, n(%) | 12 (22.6) | 17 (23.3) |  |
| No chest X-Ray/no pathological finding | 35 (66.0) | 51 (69.9) |  |
